# Purifying selection enduringly acts on the sequence evolution of highly expressed proteins in *Escherichia coli*

**DOI:** 10.1093/g3journal/jkac235

**Published:** 2022-09-08

**Authors:** Atsushi Shibai, Hazuki Kotani, Natsue Sakata, Chikara Furusawa, Saburo Tsuru

**Affiliations:** Center for Biosystems Dynamics Research (BDR), RIKEN, Osaka 565-0874, Japan; Center for Biosystems Dynamics Research (BDR), RIKEN, Osaka 565-0874, Japan; Center for Biosystems Dynamics Research (BDR), RIKEN, Osaka 565-0874, Japan; Center for Biosystems Dynamics Research (BDR), RIKEN, Osaka 565-0874, Japan; Universal Biology Institute, School of Science, The University of Tokyo, Tokyo 113-0033, Japan; Universal Biology Institute, School of Science, The University of Tokyo, Tokyo 113-0033, Japan

**Keywords:** protein sequence evolution, E–R anticorrelation, experimental evolution

## Abstract

The evolutionary speed of a protein sequence is constrained by its expression level, with highly expressed proteins evolving relatively slowly. This negative correlation between expression levels and evolutionary rates (known as the E–R anticorrelation) has already been widely observed in past macroevolution between species from bacteria to animals. However, it remains unclear whether this seemingly general law also governs recent evolution, including past and de novo, within a species. However, the advent of genomic sequencing and high-throughput phenotyping, particularly for bacteria, has revealed fundamental gaps between the 2 evolutionary processes and has provided empirical data opposing the possible underlying mechanisms which are widely believed. These conflicts raise questions about the generalization of the E–R anticorrelation and the relevance of plausible mechanisms. To explore the ubiquitous impact of expression levels on molecular evolution and test the relevance of the possible underlying mechanisms, we analyzed the genome sequences of 99 strains of *Escherichia coli* for evolution within species in nature. We also analyzed genomic mutations accumulated under laboratory conditions as a model of de novo evolution within species. Here, we show that E–R anticorrelation is significant in both past and de novo evolution within species in *E. coli*. Our data also confirmed ongoing purifying selection on highly expressed genes. Ongoing selection included codon-level purifying selection, supporting the relevance of the underlying mechanisms. However, the impact of codon-level purifying selection on the constraints in evolution within species might be smaller than previously expected from evolution between species.

## Introduction

Is there any general law that governs the evolution of protein sequences on Earth? The rate of protein sequence evolution differs between genes. Many factors other than functional importance have been proposed as determinants for the rate of evolutionary diversification among a protein sequence, as reviewed by Zhang and Yang (2015). Among these factors, gene expression levels might be a general determinant (Krylov *et al.* 2003; Rocha and Danchin 2004; Drummond and Wilke 2008). Comparative genomics of orthologous genes of closely related species revealed a pervasive negative correlation between gene expression level and the rate of evolutionary diversification in a protein sequence, namely E–R (expression–evolutionary rate) anticorrelation (Pál *et al.* 2001). The mechanism underlying E–R anticorrelation remains unclear (Usmanova *et al.* 2021) but can be explained using the different targets of purifying selection, such as mistranslation and protein misfolding (Akashi 1994; Drummond *et al.* 2005; Drummond and Wilke 2008, 2009; Cherry 2010a; Yang *et al.* 2010; Geiler-Samerotte *et al.* 2011), incorrect and slow translation (Akashi and Gojobori 2002; Cherry 2010b; Gout *et al.* 2010; Park *et al.* 2013; Yang *et al.* 2014), and protein misinteraction (Zhang *et al.* 2008; Levy *et al.* 2012; Yang *et al.* 2012). Purifying selection is believed to be strong for highly expressed proteins because the defects in the quality and quantity of these proteins presumably confer more deleterious effects on the cells than those of poorly expressed proteins.

The ubiquity of E–R anticorrelation in evolution between species is well known. However, whether the same law governs evolution within species, including past and de novo evolution, in some organisms, remains unknown. Interestingly, the advent of genomic sequencing and high-throughput phenotyping has revealed several gaps between the 2 evolutionary processes, particularly among bacteria. Notably, bacterial phenotypic diversification in nature is biphasic, whereby phenotypic diversification (such as metabolism) occurs rapidly and instantaneously within species, while divergence between species or genera proceeds gradually (Plata *et al.* 2015). Consistent with this general trend in phenotypes, recent studies have also revealed an unexpectedly large genetic divergence of protein sequences attributable to weaker purifying selection within bacterial species in natural ecosystems (Garud *et al.* 2019; Ramiro *et al.* 2020). In particular, Garud *et al.* (2019) reported that the purifying selection for protein sequences within species is much weaker than that between species, suggesting a cautionary note for the applicability of the E–R anticorrelation in relatively recent evolution among bacteria. In addition, recent studies have pointed out the inconsistency between diverse empirical data across multiple organisms and the predictions from the frequently suggested possible mechanisms explaining the E–R anticorrelation (Plata *et al.* 2010; Plata and Vitkup 2018; Razban 2019; Usmanova *et al.* 2021). For instance, recent genome-scale data empirically measuring protein stability, protein aggregation, and protein stickiness do not support the considerable extent of selection against protein misfolding or protein misinteraction for highly expressed proteins in *Escherichia coli* (Usmanova *et al.* 2021). In turn, these conflicts raise questions about the generality of the E–R anticorrelation and the relevance of the plausible mechanisms governing it, which motivated us to test the applicability of E–R anticorrelation on bacterial evolution within species and the relevance of the possible underlying mechanisms. In fact, several studies have examined whether E–R anticorrelation occurs in recent evolution within species in different organisms (Liu *et al.* 2008; Slotte *et al.* 2011; Alvarez-Ponce *et al.* 2016, 2019). These studies reported evidence implying the existence of E–R anticorrelation at the polymorphism level. Nevertheless, the numbers of mutations accumulated during evolution within species, including past and de novo evolution, are generally so small that diverse strains are often required to obtain reliable estimations of the rates of sequence evolution within species for individual genes. Similarly, the reliable estimation of the expression levels of each gene requires a large dataset obtained under various conditions because the expression levels are condition dependent. In bacteria, estimations of their evolutionary rates have been mostly based on few strains, and some transcriptome data have been used to estimate the expression levels (Petersen *et al.* 2007; Alvarez-Ponce *et al.* 2016; Feugeas *et al.* 2016). Thus, comprehensive datasets for both the genome and transcriptome are required to obtain a representative evaluation of the E–R anticorrelation within species.

To this end, we analyzed the genome sequences of 99 strains of *E. coli*, whose mutations accumulated through evolution within species in nature. We also explored the E–R anticorrelation of de novo evolution via an evolution experiment using *E. coli.* We found significant E–R anticorrelation in both past and de novo evolution in *E. coli*. We also found that purifying selection acting on highly expressed genes contributed to the ubiquity of the E–R anticorrelation. This study confirmed that purifying selection acting on highly expressed genes is not an evolutionary legacy but rather an active component, implying that expression level has a ubiquitous impact on the speed of evolutionary molecular diversification in bacteria. The detected selection included codon-level purifying selection, which supports the relevance of the underlying mechanisms proposed previously. Nevertheless, their effects on recent evolution may be smaller than expected. Our study emphasizes the importance of the expression level in understanding how genetic divergence emerges within a bacterial species and also provides new insight into the controversy of the dominant mechanisms underlying the E–R anticorrelation.

## Materials and methods

### Database analysis of mRNA expression levels

A total of 218 microarray datasets of *E. coli* K-12 substrain MG1655 with the GPL3154 platform were used in this study (Supplementary Table 1). They were included in 27 experiments and downloaded from the Gene Expression Omnibus (Barrett *et al.* 2013). After quantile normalization (Bolstad *et al.* 2003), the average and variance of the expression levels were calculated for each gene.

### Interspecific analysis of protein evolution

The protein evolutionary rates of *E. coli* were obtained from the literature, which compared the genomes of *E. coli* K-12 MG1655 and *Salmonella typhimurium* LT2 (“Supplementary information S2” in Zhang and Yang 2015). The d*N* and d*S* values were calculated from the genomic sequences of *E. coli* str. K-12 substr. MG1655 and *Salmonella enterica* subsp. *enterica* serovar Typhimurium str. LT2 (accession no. NC_000913.3 and NC_003197.2). A total of 3,145 paired sets of orthologous genes were detected by the bidirectional best hits (Overbeek *et al.* 1999) method, comparing all combinations of 2 coding features from the genomes. For each orthologous gene set, Clustal Omega (McWilliam *et al.* 2013) was used to generate an alignment, and PAML was used to calculate the d*N* and d*S* values from the alignment (Yang 1997).

### Intraspecific d*N*/d*S* analysis

The coding DNA sequences for 99 *E. coli* genomes were downloaded from Ensembl Genomes (Zerbino *et al.* 2018) in the multi-fasta format (Supplementary Table 2). Each coding feature of the genomes was annotated by the bidirectional best hits (Overbeek *et al.* 1999) method compared with the *E. coli* K-12 substrain MG1655, generating groups of orthologous genes. For each orthologous gene set, Clustal Omega (McWilliam *et al.* 2013) was used to generate an alignment, and the subfunction, “Phylogeny,” from Clustal W2 was utilized to generate a phylogenetic tree from the alignment with the neighbor joining (NJ) method. Furthermore, PAML (Yang 1997) was used to calculate the d*N* and d*S* values for each tree. Thus, we constructed a single tree for each gene in strain MG1655 along with orthologs obtained from other 98 strains and calculated the d*N*/d*S* for each single tree. We used the Clustal tools for the convenience of computing resources and equipment. To confirm the robustness of the computed evolutionary rates, we also constructed the phylogenetic tree for a subset of genes (100 randomly selected genes) based on a maximum likelihood method using RAxML (Stamatakis 2014). The trees were then used to compute the evolutionary rates (d*N*_wth_, d*S*_wth_ and d*N*_wth_/d*S*_wth_). In any cases, we confirmed a good agreement between the 2 methods as detailed in Supplementary Fig. 2.

### Strain and culture conditions

We used the *E. coli* K12 substrain MDS42 (Pósfai *et al.* 2006) as the ancestor of the evolution experiment. We used a chemically defined medium, mM63, which comprised 62 mM K_2_HPO_4_, 39 mM KH_2_PO_4_, 15 mM (NH_4_)_2_SO_4_, 2 µM FeSO_4_·7H_2_O, 15 µM thiamin hydrochloride, 203 µM MgSO_4_·7 H_2_O, and 22 mM glucose (Kashiwagi *et al.* 2009). The cells were inoculated into 8 mL of the mM63 medium and incubated with shaking at 37°C.

### Evolution experiment

The evolution experiment procedure consisted of a 4-day cycle of a serial transfer cycle. We used an automated UV-irradiating cell culture system that was previously reported (Shibai *et al.* 2019). First, the optical density (OD) value of the cell culture was measured automatically. When the OD value exceeded the stipulated threshold (OD_THR_), the cells were exposed to a dose of UV light which killed the cells, resulting in a survival rate of the ancestral cell of 10^−2^ to 10^−3^. Then, the threshold, OD_THR_, was renewed as OD_THR_ + OD_STEP_, so that the next UV irradiation was conducted when the living cell population recovered to the amount corresponding to OD_STEP_. The OD_STEP_ and initial OD_THR_ values were set at OD_600_ = 0.0015. The cells were glycerol stocked at the end of each round.

### Whole-genome resequencing

Cells were grown in a mM63 medium at 37°C with shaking at 200 rpm overnight for 2 days, which were then pelleted by centrifugation. Genomic DNA was extracted from the cells using a Wizard Genomic DNA Purification Kit (Promega). DNA libraries were prepared using a Nextera XT kit (Illumina) for paired-end sequencing (2 × 300 bp), according to the manufacturer’s instructions. Illumina MiSeq sequenced the DNA libraries using the MiSeq Reagent Kit v3 for 600 cycles. Mutation detection was performed by mapping the resulting read data to the reference genome sequence (accession no. AP012306.1) using the Burrows-Wheeler Aligner software (Li and Durbin 2009) and SAMtools (Li *et al.* 2009). For quality control, the called mutations were filtered using the Phred quality score (Ewing and Green 1998; Cock *et al.* 2010) with a cutoff value of >100. In addition, base-pair substitutions (BPSs) with a frequency of “mutant” reads <90% were removed. The resulting mutations were annotated using an in-house program written in C++.

### Calculation of d*N* and d*S* in de novo evolution

Genome-wide d*N*/d*S* values were calculated from the numbers of both synonymous and nonsynonymous BPSs using a previously reported method (Shibai *et al.* 2017). Briefly, d*N* was calculated as the number of nonsynonymous BPSs divided by nonsynonymous sites, which were normalized to codon usage and the probability of each substituted codon being nonsynonymous. d*S* was calculated in the same way using synonymous BPSs. d*N* and d*S* values in de novo evolution for each gene, referred to as d*N*_novo_ and d*S*_novo_, were calculated similarly, considering each gene sequence as a full-length sequence.

### Calculation of the factors to be controlled for each gene

In the partial correlation analysis of E–R anticorrelation, we assigned the following information to each gene of MG1655. Gene dispensability is the maximal growth rate of gene deletion mutants obtained from Campos *et al.* (2018). Gene essentiality indicates whether the gene defect results in zero growth (Goodall *et al.* 2018). Gene duplicability indicates whether the gene is a singleton or duplicated gene (i.e. paralogs). Paralogs were identified using the *E. coli* Genome Project (https://www.genome.wisc.edu/functional/paralog.htm), while singletons were defined as proteins that did not show sequence similarity to any other proteins. The number of protein–protein interactions (i.e. PPI degree) was obtained from Zitnik *et al.* (2019).

### Calculation of *G* scores

The *G* score was defined as the actual number of mutations (*M*) multiplied by the logarithm of the ratio of the actual number of mutations to the expected number of mutations (log(*M*/*E*)) (Tenaillon *et al.* 2016). Therefore, the *G* score was supposed to show positive values with mutationally accelerated genes, negative values with suppressed genes, and zero values with nonbiased genes. In this study, we normalized the *G* score by the number of mutational sites in each gene for more precise bias analyses. Specifically, the *G* score of each gene for synonymous (subscripted with *S*) and nonsynonymous (subscripted with *N*) substitutions were calculated according to the following formulas:

Normalized *G* score of synonymous and nonsynonymous substitutions of gene i:

$$\begin{array}{c} G_{S,i}=\frac{M_{S,i}}{L_{i}P_{S,i}}\ln\left[\frac{M_{S,i}}{E_{S,i}}\right]\\ G_{N,i}=\frac{M_{N,i}}{L_{i}\left(1-P_{S,i}\right)}\ln\left[\frac{M_{N,i}}{E_{N,i}}\right] \end{array}$$

Expected number of synonymous and nonsynonymous substitutions of gene i:

$$\begin{array}{c} E_{S,i}=\frac{L_{i}P_{S,i}}{〈P_{S}〉}\frac{\sum_{i}^{K}M_{S,i}}{\sum_{i}^{K}L_{i}}\\ E_{N,i}=\frac{1-P_{S,i}}{P_{S,i}}E_{S,i} \end{array}$$

$M_{S,i}$ is the observed number of synonymous substitutions in gene i. $M_{N,i}$ is the observed number of nonsynonymous substitutions in gene i. K is the number of genes in the genome. $L_{i}$ is the length of the coding DNA sequence of gene i. $P_{S,i}$ is the probability that the substitution is synonymous substitution when a substitution occurs in gene i as detailed below. $〈P_{S}〉$ represents the mean of $P_{S,i}$ for all the genes.

The probability that the substitution occurred on a given codon when a substitution occurred in gene i was calculated using the following equation:

$$P\left({\mathrm{cod}}_{k,i}|{\mathrm{sub}}_{j}\right)=\frac{P\left({\mathrm{cod}}_{k,i}\right)n({\mathrm{sub}}_{j}{|\mathrm{cod}}_{k,i})}{\sum_{x=1}^{64}P\left({\mathrm{cod}}_{x,i}\right)n({\mathrm{sub}}_{j}{|\mathrm{cod}}_{x,i})}.$$

Here, each substitution of all 6 possible substitutions is denoted by ${\mathrm{sub}}_{j}$, where j takes 1–6, using the following array:

sub=(AT→TA, GC→CG,AT→GC, AT→CG,GC→AT, GC→TA).

In addition, each codon of all 64 possible codons in a given gene i is denoted by ${\mathrm{cod}}_{k,i}$, where k takes 1–64, using the following array:

cod=(AAA, AAT, AAG, …, CCC).

The codon usage of codon k in gene i is then represented by $P\left({\mathrm{cod}}_{k,i}\right)$, which was calculated from the genome sequence of the ancestral strain. In addition, the number of possible mutant triplets when the jth substitution occurs in a given ${\mathrm{cod}}_{k}$ in gene i is denoted by$\;n({\mathrm{sub}}_{j}{|\mathrm{cod}}_{k,i})$. Therefore, the probability of synonymous change for a given codon in gene i with a given j th substitution is given by the following equation:

$$P\left({S|{\mathrm{sub}}_{j}\cap \mathrm{cod}}_{k,i}\right)=\frac{n({S|{\mathrm{sub}}_{j}\cap \mathrm{cod}}_{k,i})}{n({\mathrm{sub}}_{j}{|\mathrm{cod}}_{k,i})}.$$

Here, the number of synonymous triplets when a ${\mathrm{sub}}_{j}$ occurs in a given ${\mathrm{cod}}_{k,i}$ is denoted by $n({S|{\mathrm{sub}}_{j}\cap \mathrm{cod}}_{k,i})$. Using the mutational spectrum for synonymous substitutions, $P\left({\mathrm{sub}}_{j}\right)$, these 2 probabilities give $P_{S,i}$ using the following equation:

$$P_{S,i}=\sum_{j=1}^{6}\left\{P\left({\mathrm{sub}}_{j}\right)\sum_{k=1}^{64}\left[P({\mathrm{cod}}_{k,i}|{\mathrm{sub}}_{j})P\left({S|{\mathrm{sub}}_{j}\cap \mathrm{cod}}_{k,i}\right)\right]\right\}.$$

### Calculation of the codon adaptation index

The codon adaptation index (CAI) indicates the abundance of optimal codons in a gene sequence, where an optimal codon is defined as the most frequent codon in each of the synonymous codon groups used in the most abundant proteins (Sharp and Li 1987). The CAI of a given gene with an amino acid length La was calculated as follows:

$$\begin{array}{c} \mathrm{CAI}={\left(\prod_{j}^{La}\frac{f_{j}}{\max[f_{k}]}\right)}^{\frac{1}{La}}\;j,k\\ \in \left[\mathrm{synonymous}\;\mathrm{codons}\;\mathrm{for}\;\mathrm{amino}\;\mathrm{acid}\right] \end{array}$$

where $f_{j}$ is the frequency of the codon coding for jth amino acid of the given gene and $\max[f_{k}]$ represents the frequency of the most frequent synonymous codon $f_{k}$ for that amino acid. We calculated the frequency of each codon by considering the 40 most abundant genes based on the transcriptome of the ancestral strain.

### Calculation of *C* score

The *C* score is an indicator of bias in codon weight change caused by a synonymous substitution. Note that the *C* score was calculated for each mutation, not for each gene, as in the other indicators used in this study. The *C* score in which an ancestral codon (a) changes to a mutated codon (m), referred to as $C_{a\to m}$, is defined as follows:

$$C_{a\to m}=\ln\left[w_{m}\right]-W_{a}$$

where $w_{m}$ is the codon weight of codon m calculated by the following formula:

$$w_{m}=\frac{f_{m}}{\max[f_{k}]}$$

where $f_{m}$ is the frequency of codon m of the focal amino acid and $\max[f_{k}]$ represents the frequency of the most frequent synonymous codon $f_{k}$ for that amino acid. In addition, $W_{a}$ is the average of the logarithms of the codon weights with a single synonymous substitution of codon a and corresponds to the expected value of the mutated codon weights as follows:

$$W_{a}=\frac{1}{\sum_{n\in S_{a}}P_{a\to n}}\sum_{n\in S_{a}}P_{a\to n}\ln\left[w_{n}\right].$$

$S_{a}$ is the set of all possible synonymous codons from a given ancestral codon a by a single BPS, $m\in S_{a}$. $P_{a\to n}$ is the frequency of a BPS that enables synonymous mutation from codon a to codon n, which was calculated by the mutational spectrum of synonymous substitutions.

### Gene ontology analysis

Gene ontology (GO) enrichment analysis was performed using GOstats (v.2.48.0, R Bioconductor) (Falcon and Gentleman 2007). We used all 3 categories: biological process (BP), molecular functions (MF), and cellular components (CC). The resulting GO terms were filtered with cutoffs of 0.01 and 0.05 for their respective *P*-value and *q*-value (Storey *et al.* 2021). Genes within the top and bottom 10% of the normalized *G* score were analyzed as gene sets. For visualization, the detected GO terms were converted to their ancestral GO terms in the second level of the GO tree, that is, the layers directly under BP, MF, or CC.

### mRNA expression profiling of genes using microarray technology

The cells were cultured for 16–19 h and then sampled at the time of the logarithmic growth phase (OD_600_ values were 0.072–0.135). Aliquots of the cells were immediately added to the same volume of ice-cold ethanol containing 10% (w/v) phenol. RNA extraction was performed using a RNeasy mini kit with on-column DNase digestion (Qiagen), following the manufacturer’s protocol. The purified RNA was quality-controlled using an Agilent 2100 Bioanalyzer and an RNA 6000 Nano kit (Agilent Technologies). A microarray experiment was performed using an Agilent 8 × 60 K array, which was designed for the *E. coli* W3110 strain so that 12 probes were contained for each gene. Purified total RNA (100 ng) was labeled with Cyanine3 (Cy3) using a Low Input Quick Amp WT labeling kit (One-color; Agilent Technologies). The Cy3-labeled cRNA was checked for its amount (>5 µg) and specific activity (>25 pmol/µg) using NanoDrop ND-2000. Then, the cRNA of 600 ng was fragmented and hybridized to a microarray for 17 h at 65°C, rotating at 10 rpm in a hybridization oven (Agilent Technologies). The microarray was then washed and scanned according to the manufacturer’s instructions. Microarray image analysis was performed using Feature Extraction version 10.7.3.1 (Agilent Technologies). The resulting gene expression levels were normalized using quantile normalization.

## Results

### The inter- and intraspecific E–R anticorrelation in past evolution

**Fig. 1.**
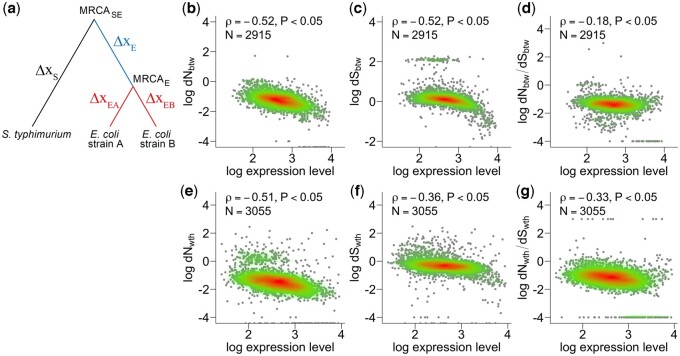
The negative correlation between mRNA expression level and the rates of DNA sequence of orthologs in the course of past evolution. a) A schematic phylogeny of *E. coli* and *S. typhimurium.* Genetic changes between nodes are indicated as Δx_S_ for *S. typhimurium* from the last common ancestor of *E. coli* and *S. typhimurium* (LCA), Δx_E_ for the most recent common ancestor of *E. coli* (MRCA) from the LCA, Δx_EA_, and Δx_EB_ for *E. coli* strains A and B from the MRCA, respectively. Genetic changes between species, N_btw_ and S_btw_ included, represent the difference between Δx_S_ and the sum of Δx_E_ and Δx_EA_ or the sum of Δx_E_ and Δx_EA_. Genetic changes within *E. coli* species, N_wth_ and S_wth_ included, represent differences between Δx_EA_ and Δx_EB_. b–d) The negative correlation of the rate of interspecific evolution of DNA sequences (*E. coli* and *S. typhimurium*). e–g) The negative correlation of the rate of intraspecific evolution of DNA sequences (*E. coli*). The evolutionary rate of the DNA sequence is characterized by d*N* (b and e) and d*S* (c and f), respectively. The d*N*/d*S* ratio of interspecific (d) and intraspecific evolution (g). The expression level was calculated from *E. coli* transcriptome data. Each dot corresponds to a single gene. The red–green gradient represents the 2D density (high to low). Spearman’s rank correlation coefficients and *P*-values are shown.

The rate of interspecific evolution among protein sequences can be accounted for by the ratio between the number of nonsynonymous nucleotide changes per nonsynonymous site (d*N*) and the number of synonymous nucleotide changes per synonymous site (d*S*) in the orthologous genes between closely related species (Fig. 1a). We refer to interspecific d*N* and d*S* as d*N*_btw_ and d*S*_btw_, respectively. Previous studies have shown that both d*N*_btw_ and d*S*_btw_ are negatively correlated with expression levels in *E. coli* (Spearman’s rank correlation, ρ = −0.52 for d*N*_btw_ and ρ = −0.52 for d*S*_btw_, Fig. 1, b and c) and other organisms (Drummond and Wilke 2008). In this study, we calculated the d*N*_btw_ and d*S*_btw_ of *E. coli* by comparing it with *S. typhimurium*. The underlying mechanisms of these relationships are explained by purifying selection at the codon level (Drummond and Wilke 2008; Yang *et al.* 2010; Park *et al.* 2013). In particular, the protein misfolding avoidance hypothesis (Yang *et al.* 2010) explains that optimal codons are favored in highly expressed proteins to avoid toxic misfolding and that d*N*_btw_ and d*S*_btw_ are common rather than independent targets of codon-level purifying selection to combat misfolding. Consistent with this hypothesis, we found a negative correlation between d*N*_btw_/d*S*_btw_ and the expression level in *E. coli* (with *S. typhimurium* for d*N*_btw_/d*S*_btw_). The correlation was somewhat weaker than the E–R anticorrelation in d*N*_btw_, most likely due to the fact that the common purifying selection acting on d*N*_btw_ and d*S*_btw_ was canceled out (ρ = −0.18, Fig. 1d). Nevertheless, the negative correlation between d*N*_btw_/d*S*_btw_ and the expression level remains substantial, suggesting that another mechanism contributes to purifying selection, which acts on highly expressed genes.

To test whether within-species molecular evolution also follows the E–R anticorrelation, we quantified intraspecific d*N* and d*S*, referred to as d*N*_wth_ and d*S*_wth_, among 99 strains of *E. coli*. We found that both d*N*_wth_ and d*S*_wth_ were negatively correlated with gene expression relative to interspecific evolution (ρ = −0.51 for d*N*_wth_ and ρ = −0.36 for d*S*_wth_, Fig. 1, e and f). In addition, the correlation coefficient for d*N*_wth_ was slightly larger than that for d*S*_wth_, which is in agreement with the genetic signatures of interspecific evolution in other organisms, such as yeast or flies. This difference between d*N*_wth_ and d*S*_wth_ also suggests that the E–R anticorrelation in d*N*_wth_ reflects purifying selection in targets different from those in d*S*_wth_, as in the case of the E–R anticorrelation in d*N*_btw_. To confirm this hypothesis, we explored the relationship between d*N*_wth_/d*S*_wth_ and expression levels. As with the case of interspecific evolution, d*N*_wth_/d*S*_wth_ showed a substantial negative correlation with expression level, although the correlation was weaker than the E–R anticorrelation in d*N*_wth_. Therefore, the purifying selection on d*S*_wth_ seems to be insufficient to explain the E–R anticorrelation in intraspecific evolution. These results suggest that E–R anticorrelation itself might be causal to a general pattern of molecular evolution in the past, but the underlying mechanisms of purifying selection remain an open question, as stated recently in the literature (Plata and Vitkup 2018).

### E–R anticorrelation in de novo evolution

**Fig. 2.**
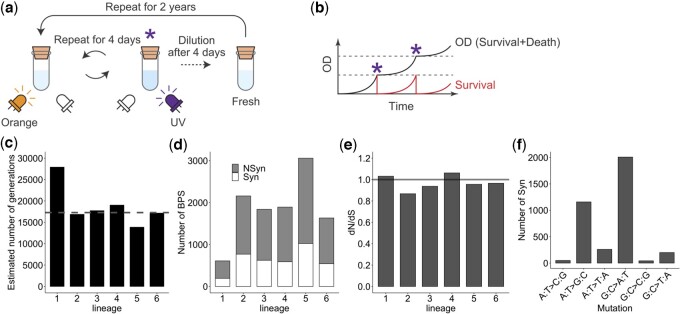
Evolution experiment for accumulating massive mutations. a) Procedure of an evolution experiment with the UV-irradiating cell culture device. The device consists of a quartz glass test tube with a resin housing that measures the cell density (OD) by an orange LED and irradiates UV light by a UV-C LED. Mutagenesis by UV irradiation (denoted as asterisks) was performed when OD exceeded a defined increment so that the survival fraction could be maintained within a constant range (b). After 4 days of repeats, an aliquot of cell culture was diluted with fresh media 100 times and transferred into a new test tube. These procedures were repeated for 6 independent replicates for 2 years. c) The estimated number of generations after 688 days of the evolution experiments. The black bars correspond to the values calculated with the doubling time of evolved cells for each of the 6 replicates. The dashed line indicates the value calculated with the ancestral doubling time. d) The number of accumulated BPSs during the evolution experiment. The gray and white fractions of a bar represent nonsynonymous and synonymous substitutions, respectively. e) The genome-wide d*N*/d*S* values were close to 1.0 for all the 6 replicates, implying that the majority of the accumulated mutations had neutral effects on their fixation within the populations. f) Mutation spectrum of synonymous substitutions. The synonymous substitutions of all lineages are summed for each substitution type.

To determine whether the E–R anticorrelation is an evolutionary legacy or is currently applicable, we explored the relationship between protein evolutionary speed and gene expression levels during de novo evolution. Using a previously developed UV-irradiating cell culture device (Shibai *et al.* 2019), we conducted an evolution experiment to rapidly accumulate mutations (Fig. 2a). *E. coli* cells were incubated in this device and transferred to a fresh medium every 4 days. During incubation, the device automatically measured the OD of the culture and irradiated UV for each unit increment of OD, where UV was utilized as a mutagen and germicidal lamp (Fig. 2b). This feedback control of UV irradiation prevented the depression of mutation rates caused by the acquisition of UV resistance in the cells. We established 6 independent lineages from an ancestral colony and repeated the cycle of incubation and transfer for 2 years, corresponding to tens of thousands of generations (Fig. 2c). As a result, we obtained thousands of BPSs of the coding region fixed in each cell population (Fig. 2d). The occurrence of the same mutations over multiple lineages was exceedingly rare, ensuring that most of the accumulated BPSs contributed to the evolutionary diversification of the DNA sequence. To understand the overall evolutionary processes of diversification, we calculated whole-genome d*N*/d*S* values (Fig. 2e) by considering a mutational spectrum (Fig. 2f). The d*N*/d*S* of most lineages was roughly 0.9, indicating that most BPSs were fixed in the populations through neutral processes rather than by adaptive processes. Moreover, considering the large population size and high mutation rate in the culture device, many of these nonsynonymous BPSs were likely to be fixed in the population by hitchhiking rather than genetic drift.

**Fig. 3.**
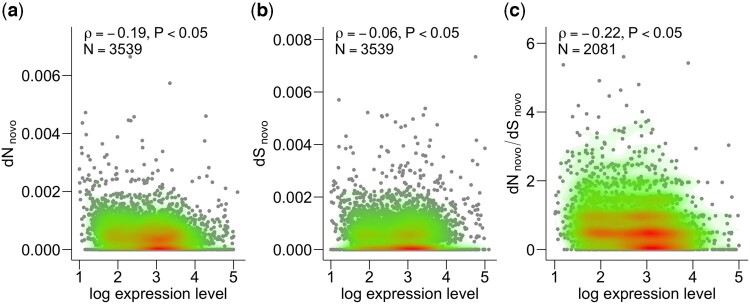
There was a negative correlation between the protein sequence evolution during the evolution experiment and the gene expression level. a) d*N*_novo_ showed a negative correlation with the gene expression level. b) On the other hand, d*S*_novo_ showed only a slight correlation with the expression level. c) A negative correlation was also observed for d*N*_novo_/d*S*_novo_, where d*N*_novo_ was normalized by d*S*_novo_ by canceling the common selection.

To explore the expression levels of the mutated genes, we obtained transcriptome data of the ancestral and evolved samples by microarray and quantified the geometric mean of 6 independent lineages. We found that the expression profiles of the evolved strains were similar to that of the ancestral strain (ρ = 0.89–0.94, Supplementary Fig. 1). Using transcriptome data, we explored the relationship between the protein evolutionary rate and gene expression levels during de novo evolution. For each gene, we quantified d*N* and d*S* in de novo evolution, referred to as d*N*_novo_ and d*S*_novo_, by using the sum of the number of nonsynonymous and synonymous BPSs among 6 independent lineages. We found significant E–R anticorrelation even in de novo evolution, with both ancestral (ρ = −0.17, *P* < 0.05) and evolved expression levels (ρ = −0.19, *P* < 0.05, Fig. 3a). We also confirmed that this negative correlation remained after controlling for gene dispensability (ρ = −0.18), gene essentiality (ρ = −0.17), gene duplicability (ρ = −0.19), and number of protein–protein interactions (ρ = −0.16) as confounding variables (partial correlation tests, see *Materials and Methods*). Notably, the mutation data of approximately half the number of total mutations (i.e. the data at 1 year of evolution) exhibited a similar negative correlation (ρ = –0.16, *P* < 0.05). Thus, we confirmed that the observed E–R anticorrelation was relatively weak but insensitive to the progress of our evolution experiment or to changes in transcription profiles, at least during our evolution experiment. Contrary to the evolution between species, the negative correlation between d*S*_novo_ and expression levels was found to be much weaker than that between d*N*_novo_ and expression levels (ρ = −0.06, Fig. 3b). We also confirmed a negative correlation between d*N*_novo_/d*S*_novo_ and expression levels (ρ = −0.22, Fig. 3c) as well as the E–R anticorrelation in d*N*_novo_. Thus, our de novo evolution experiments revealed ongoing purifying selection on highly expressed genes.

### Purifying selection on codon usage in de novo evolution was less sensitive to expression level

**Fig. 4.**
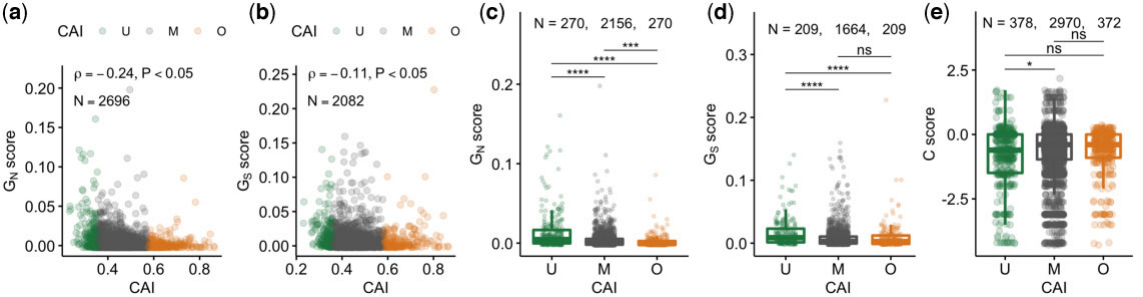
Relation between *G* scores and CAI. The CAI was negatively correlated with *G* scores for nonsynonymous (*G_N_*, a) and synonymous BPSs (*G_S_*, b). Spearman’s rank correlation and *P*-values are indicated in each panel. Color represents codon optimality (U, unoptimized; M, moderate; O, optimized proteins). Comparison between codon optimality and *G* scores (*G_N_*, c; *G_S_*, d). Enlarged panels are shown at the bottom. e) Comparison between codon optimality and *C* score. Adjusted *P*-values for Wilcoxon test are indicated as ns >0.05, *<0.05, ***<0.001, and ****<0.0001.

The expression level dependency of d*S* reflects the purifying selection of codon usage of highly expressed proteins, which is a frequently suggested explanation for the E–R anticorrelation in d*N* (Drummond and Wilke 2008). Highly expressed proteins tend to use optimal codons that enable fast and accurate translation (Akashi 2001, 2003) and protein stability (Yang *et al.* 2010). The use of other unfavorable codons has detrimental effects on cellular growth and is thought to be evolutionarily constrained (Zhang and Yang 2015). However, the small anticorrelation between d*S*_novo_ and expression levels obscures the expected expression dependency of the purifying selection on codon usage in de novo evolution. To clarify this, we explored the relationship between the degree of codon optimization of each protein and the evolutionary speed of synonymous BPSs. Since this relationship is expected to be weak, it is important to evaluate the evolutionary speed of a small number of synonymous BPSs. To this end, we used a normalized version of the *G* score, hereinafter referred to as the *G* score, as an alternative to d*N*_novo_ and d*S*_novo_, as detailed in the *Materials and Methods*. The *G* score is useful for screening genes with a small number of substitutions relative to neutral expectations. First, we reconfirmed the E–R anticorrelation between expression level and *G* score in nonsynonymous substitutions (*G_N_*, ρ = −0.15, *P* < 0.05) and that there was no correlation in synonymous substitutions (*G_S_*), which was consistent with the relationship between expression level and d*N*_novo_ or d*S*_novo_. Next, we employed the CAI as a standard measure of the degree of codon optimization and explored the relationship between CAI and *G* scores. As a result, a negative correlation was found between the CAI and *G* score for nonsynonymous (ρ = −0.24, Fig. 4a) and synonymous (ρ = −0.11, Fig. 4b) BPSs; however, the correlation coefficient for synonymous BPSs was not strong. To confirm the looseness of the purifying selection on codon-optimized proteins in de novo evolution, we classified 10% of mutated proteins with the lowest CAI as unoptimized, 10% of mutated proteins with the highest CAI as optimized, and the remaining mutated proteins as having moderate optimality in terms of codon usage for nonsynonymous and synonymous BPSs. As expected, unoptimized proteins showed higher *G_S_* than the optimized and moderately optimized proteins (Fig. 4d). In contrast, there was no significant difference between optimized and moderately optimized proteins, indicating that the purifying selection on codon usage only weakly depends on expression levels in de novo evolution. This tendency remained even if the classification criteria for CAI changed from 10% to 5%. To confirm the looseness of the purifying selection on codon usage more directly, we focused on individual synonymous BPSs and explored codon bias. To this end, we calculated the *C* score for synonymous BPSs, whereby the *C* score represents the difference in preference of the mutant synonymous codon from neutral expectation, as detailed in the *Materials and Methods*. In short, the *C* score takes positive values if the mutant synonymous codons are used more frequently in highly expressed proteins than in neutral expectations, while it takes negative values if the mutant synonymous codons are used less frequently in highly expressed proteins than in neutral expectations. Contrary to the statistics, such as *G* scores or CAI, characterizing each gene, *C* scores are assigned to each synonymous BPS, not to each gene. In other words, each gene had as many *C* scores as the number of synonymous BPSs in each gene. We found that unoptimized proteins allowed for more mutant synonymous codons, which are infrequently used in highly expressed proteins than moderately optimized codons (Fig. 4e). In contrast to the other categories, the mutant synonymous codons of the optimized proteins were not able to obtain high *C* scores because the wild-type codons of the optimized proteins are likely to be the most frequent among the highly expressed proteins. Therefore, it is reasonable that there were no significant differences in *C* scores between optimized and unoptimized proteins, even though the former had a relatively larger score than the latter. Altogether, these results support that the detected purifying selection on codon usage is active but less sensitive to expression levels.

### Purifying selection of synonymous substitution on molecular function

**Fig. 5.**
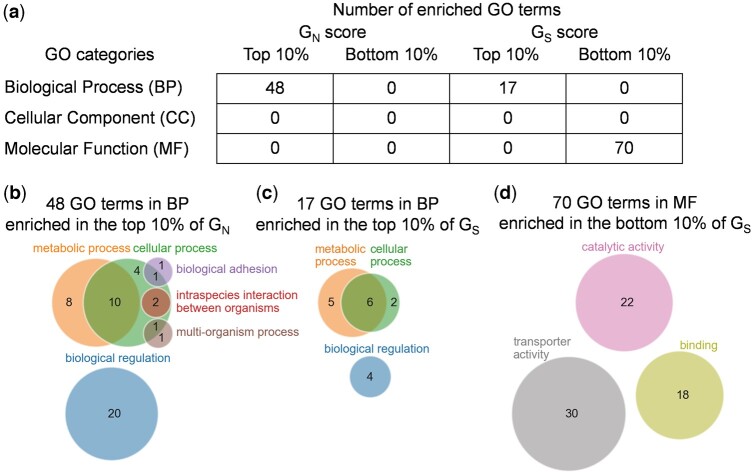
Comparison between *G* scores with biological features. a) Enrichment analysis for the top and bottom 10% of *G_N_* and *G_S_*. The number of GOs enriched significantly was shown in each class. b–d) Venn diagram of the ancestral GOs at the second level (circles) of the GO tree for each of the enriched GOs (b for top 10% of *G_N_*, c for top 10% of *G_S_*, and d for bottom 10% of *G_S_*). The number of enriched GOs in each parental GO is indicated in each circle.

The difference between d*N*_novo_ (or *G_N_*) and d*S*_novo_ (or *G_S_*) in correlation with expression levels suggests that the protein features on which purifying selection acts in de novo evolution of synonymous BPSs might be somewhat different from that of nonsynonymous BPSs. To confirm this possibility, we conducted a GO enrichment analysis for the proteins ranked in the top or bottom 10% of *G* scores for synonymous and nonsynonymous BPSs (Fig. 5). We found 70 GO terms enriched in the bottom 10% of *G_S_*; in contrast, no GO terms were enriched in the bottom 10% of *G_N_* (Fig. 5a). Interestingly, all of the enriched terms were classified in the MF category, suggesting that some enzymatic features were related to the target of purifying selection for synonymous BPSs rather than any metabolic pathways. For instance, the enriched GO terms contained ATPase activity (GO: 0016887), which is required for various biochemical reactions (Fig. 5d), regardless of metabolic pathways. Contrary to the bottom 10% of *G_S_*, the top 10% of *G_S_* showed no enrichment in the MF category; however, 17 GO terms were enriched in the BP category, such as the lipopolysaccharide biosynthetic process (GO: 0009103). Many of these were common among the GO terms enriched in the top 10% of *G_N_* (Fig. 5, b and c), suggesting that some proteins related to these processes were likely to be inactivated and were not targeted by purifying selection for both synonymous and nonsynonymous BPSs. These results are consistent with those of a scenario in which purifying selection on synonymous BPSs does not play a major role in the E–R anticorrelation in nonsynonymous BPSs, at least in de novo evolution.

## Discussion

The present study explored the impact of expression levels on the molecular evolution of bacteria. By employing comparative genomics and a laboratory-based evolution experiment, we elucidated the ubiquity of the impact of expression level on the evolutionary speed of sequence diversification. We found that the E–R anticorrelation governs not only sequence diversification between species but also within species. This finding of the ubiquity of the E–R anticorrelation is consistent with the recent analysis of genomic mutations accumulated in *E. coli* over long-term evolution experiments (Maddamsetti, 2021). In support of the core finding of the previous study, we found an anticorrelation relationship between the rate of gene evolution and the level of gene expression (mRNA or protein abundance in the previous study) for accumulated mutations in the laboratory evolution of *E. coli*. However, there are several disparities between the latter and the present study. First, the correlation coefficients between the expression level and rate of nonsynonymous mutations in the long-term evolution experiments were negative, but their magnitudes were much smaller (ρ = −0.0486 to 0.0991) than those for de novo evolution (ρ = −0.19, Fig. 3a). Second, the correlation coefficients between the expression levels and rates of synonymous mutations in the long-term evolution experiments were positive (ρ = 0.0458 to 0.094), whereas the values were negative in our de novo evolution experiment (ρ = −0.06, Fig. 3b) and natural evolution within species (ρ = −0.36, Fig. 1f). We speculate that these differences arose not only from the difference in conditions between the 2 evolution experiments but also from the difference in the analytical method used to calculate the evolutionary speeds of DNA sequences. Contrary to our study, for example, the previous study included mutations unfixed in the populations to calculate the evolutionary speeds. Accounting for unfixed mutations tends to obscure the signatures of natural selection and is likely to underestimate purifying selection. In addition, the previous study did not use d*N* or d*S* but rather employed the number of nonsynonymous or synonymous mutations per length as a measure of the rate of evolution. Accordingly, neither biased mutational spectrum nor the differences in probability of synonymous/nonsynonymous sites among genes were considered properly, which could interfere with the calculation of the evolutionary speeds for each gene. On the other hand, our method carefully treats these key factors when measuring the evolutionary speeds of DNA sequences, as detailed in the *Materials and Methods*. Thus, our data support the reliability of the E–R anticorrelations. We also found that the purifying selection acting on highly expressed genes is not a legacy but actively constrains the sequence diversification of these genes, even along a relatively short evolutionary timescale. The detected selection included purifying selection at the codon level, supporting the relevance of the possible underlying mechanisms such as selection against protein misfolding or protein misinteraction, since these frequently suggested mechanisms assert codon-level purifying selection acting on highly expressed proteins (Yang *et al.* 2010, 2012). Nevertheless, our data also suggest that the impacts of these frequently suggested possible mechanisms on recent evolution might be weaker than previously expected. These findings are consistent with those of recent studies, indicating that empirical data measuring protein stability, protein aggregation, and protein stickiness do not support the considerable impact of these frequently suggested mechanisms on the E–R anticorrelation for evolution between species (Plata *et al.* 2010; Plata and Vitkup 2018; Razban 2019; Usmanova *et al.* 2021). Therefore, the unexpected weak impacts of the frequently suggested mechanisms might be common between evolution within species and evolution between species. In conclusion, this study suggests the importance of the expression level when attempting to understand how genetic divergence emerges within a bacterial species and also provides a new insight into the controversy of the dominant mechanisms underlying the E–R anticorrelation (Zhang and Yang 2015).

In this study, E–R anticorrelation was observed in both past and de novo evolution within species. However, the negative correlation of the former is stronger than that of the latter. What does this difference mean? We speculated that the magnitude of purifying selection against protein sequences could explain this difference, since the E–R anticorrelation mainly reflects the purifying selection. We found this to be true. In our experiment, the average d*N*/d*S* of past evolution was smaller than that of de novo evolution. That is, purifying selection against protein sequences in past evolution is stronger than that of de novo evolution. Why is the purifying selection in de novo evolution relatively small, even in the presence of selection for growth/survival in our evolution experiment? There are at least 2 plausible explanations for this finding. The first possible and trivial explanation is that natural environments are more severe than those experienced in test tubes. Under our laboratory conditions, the nutrients required for growth were supplied constantly and at sufficient levels. In addition, the stress factor was limited to that from the UV alone. On the other hand, the quality and quantity of both nutrients and stressors must be different from the laboratory conditions and must change unpredictably. These severe conditions enable us to speculate that the essentiality of each gene is strong even for nonessential genes, which are characterized in relatively milder laboratory conditions. In other words, the detrimental effects of a given mutation are strong under natural conditions. Therefore, it is not difficult to imagine that a strong purifying selection governs evolution in nature. The second explanation is plausible if we consider a high mutation rate in our evolution experiment. The rate of mutation in our experimental setup was hundreds of times higher than the spontaneous mutation rate that would be experienced in nature. Therefore, neutral-to-deleterious mutations are relatively frequent. The population bottleneck in our experiment was large enough to fix these frequent deleterious mutations in a population by hitchhiking driver beneficial mutations. Therefore, the deleterious effects of a given passenger mutation are alleviated by the beneficial effects of driver mutations. As a result, purifying selection cannot purge such alleviated detrimental mutations, which yields nearly neutral values for d*N*/d*S*. These mechanisms are nonmutually exclusive. Interestingly, a high mutation rate and neutrality driven by hitchhiking are not only applicable to our artificial condition, but are also seen in more natural situations (Ramiro *et al.* 2020). Therefore, the relaxation of purifying selection due to high mutation rates may partially contribute to past divergent evolution within species. Here, in our analysis, we excluded mutations likely to be polymorphic in de novo evolution. In other words, we counted only fixed mutations. This filtering might have the effect of favoring less deleterious mutations in de novo evolution. However, we can have a similar concern with the mutations detected in the analysis for the past evolution in nature, because the isolation of *E. coli* strains as single colonies from the environment might have fixed the polymorphism in the population to which the cells originally belonged. Therefore, we believe that the differences in the treatment of polymorphisms in de novo and past evolution alone are insufficient in explaining our results.

Why is E–R anticorrelation considered to be general? Different hypotheses have been proposed to explain the underlying mechanism behind E–R anticorrelation, such as the protein misfolding avoidance or misinteraction avoidance hypotheses. However, these proposed hypotheses cannot fully explain the generality of E–R anticorrelation. Previous studies have focused on identifying the type of fundamental BPs for a mutated gene that has deleterious effects on any organism. In contrast, our results suggest the importance of robustness or conservativeness of the entire transcriptional expression pattern during evolution to explain the generality of the E–R anticorrelation. If expression levels evolve without any constraints or are highly dynamic, the E–R anticorrelation would lose its generality. The expression level of a gene is expected to change dynamically during evolution, for example, by the mutation of a corresponding transcription factor or intergenic region. In fact, an enrichment analysis detected those nonsynonymous mutations significantly accumulated transcription factors in our evolution experiment. Interestingly, however, the entire transcription level exhibited only slight changes from the ancestor even after the accumulation of thousands of mutations. As a result, an equivalent level of the E–R anticorrelation was observed in both the ancestral transcriptional data and in the evolved transcriptional data (ρ = −0.21 to −0.23). Such conservativeness among expression levels was also detected in other evolutionary experiments equipped with growth selection. For example, Ho and Zhang (2018) revealed that genetic changes more frequently reverse rather than reinforce transcriptional plastic changes in adaptation to a new environment, generally because an original transcriptional state is favored during growth selection. Transcriptome level conservation has also been observed in bacterial evolution in nature (Zarrineh *et al.* 2014; Payne and Wagner 2015; Junier and Rivoire 2016). Likewise, any compensatory mutations might restore expression levels that were altered by other harmful mutations to their original levels in our evolution experiment. Therefore, some mutations among transcriptional factors may play a role in compensatory mutations to retain their expression levels. In addition to the genetic mechanism, there are cases in which an alternative mechanism without any mutations underlies conservativeness at the expression level. For instance, Briat *et al.* (2016) proposed a network motif conferring homeostasis or the perfect adaptation of expression levels to intrinsic and extrinsic disturbances. Such mechanisms are also applicable to mutational disturbances in the expression levels. In addition, it has been pointed out that ORFs can somehow determine their own expression levels (Isalan *et al.* 2008). To understand the generality of the E–R anticorrelation, the present study sheds light on the importance of understanding the quantitative relationship between protein sequence evolution and expression evolution.

## Supplementary Material

jkac235_Supplemental_Figure_S1

jkac235_Supplemental_Figure_S2

jkac235_Supplemental_Table_S1

jkac235_Supplemental_Table_S2

## Data Availability

The raw sequence data of genome sequence analyses of the ancestral and evolved samples in this article are available in NCBI's Sequence Read Archive (SRA) under the accession numbers SRR16961197 to SRR16961208. The microarray data of the ancestral and evolved samples in this article are available in NCBI's Gene Expression Omnibus (GEO) and are accessible through GEO Series accession number GSE189008. Supplemental material is available at G3 online.
