# Supplementary material for: Purifying selection enduringly acts on the sequence evolution of highly expressed proteins in Escherichia coli
Source: G3 (Bethesda). 2022 Sep 8;12(11):jkac235. doi: 10.1093/g3journal/jkac235 (PMC9635659; doi:10.1093/g3journal/jkac235)
Supplement: jkac235_Supplemental_Figure_S1 [file jkac235_supplemental_figure_s1.pdf]

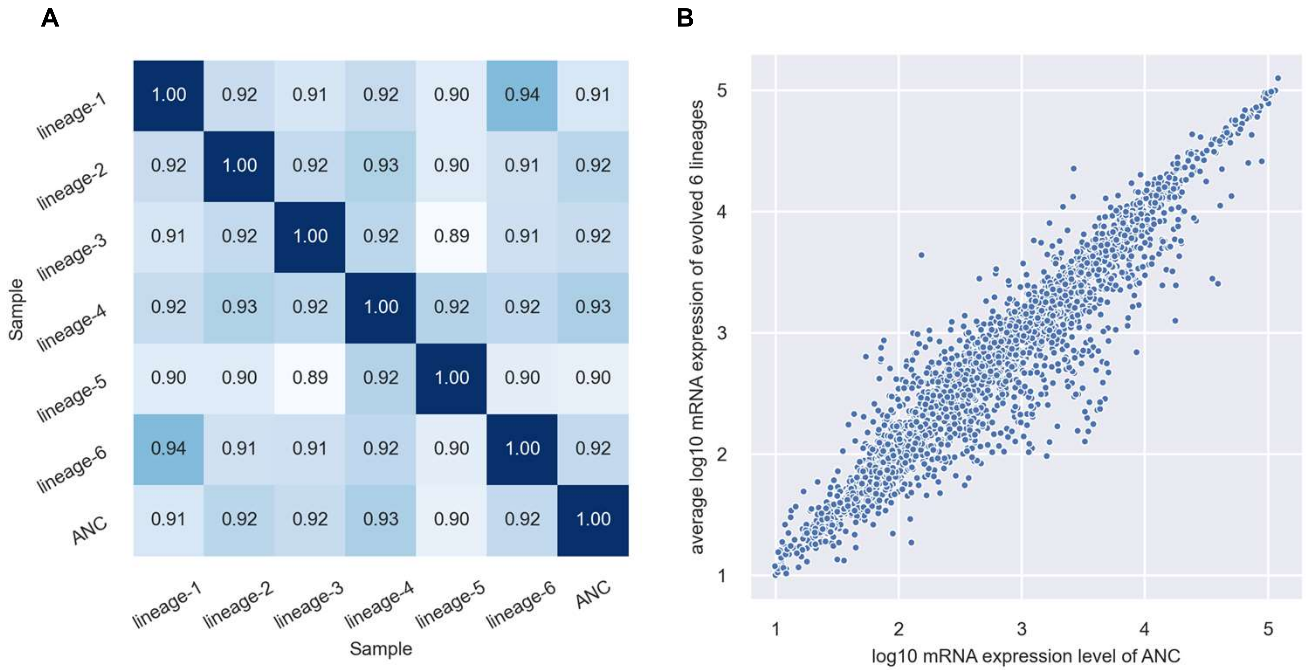

Figure S1. Gene expression levels showed similar values before and after mutation accumulation and between biological replicates. (A) The log10 transformed expression levels were compared between samples. The values in the matrix are correlation coefficients calculated with Spearman's rank correlation. Lineages 1 to 6 show the replications of mutation accumulation experiments. ANC refers to their ancestral sample. **(B)** Average of log10 transformed expression levels of 6 replicates with expression levels of their ancestral strain. The correlation coefficient of this plot is $\rho = 0.949$.
