# Supplementary material for: Purifying selection enduringly acts on the sequence evolution of highly expressed proteins in Escherichia coli
Source: G3 (Bethesda). 2022 Sep 8;12(11):jkac235. doi: 10.1093/g3journal/jkac235 (PMC9635659; doi:10.1093/g3journal/jkac235)
Supplement: jkac235_Supplemental_Figure_S2 [file jkac235_supplemental_figure_s2.pdf]

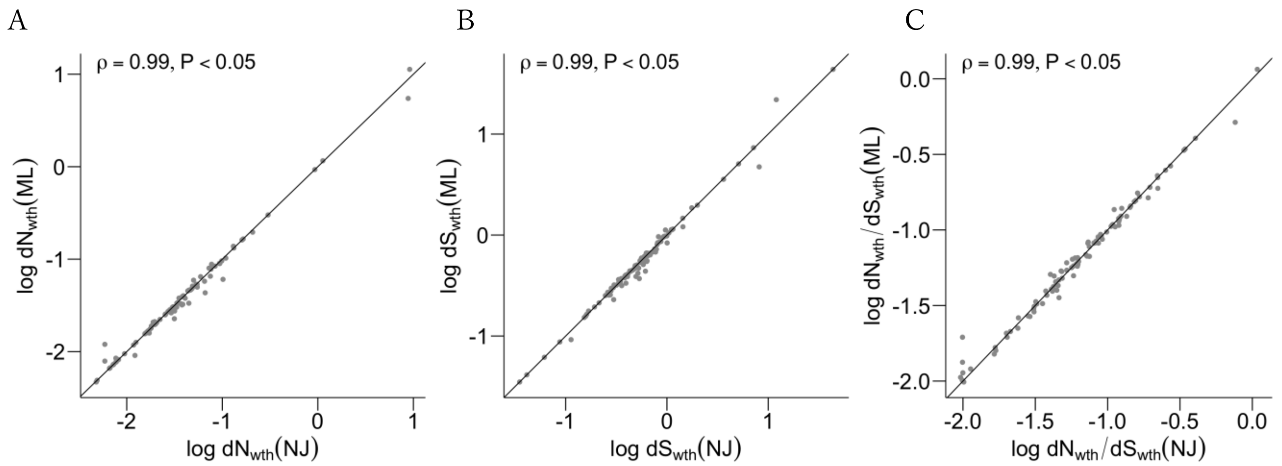

Figure S2. Comparison of the evolutionary rates between different methods of constructing phylogenetic trees. In this study, we employed the neighbor joining (NJ) method to construct a phylogenetic tree within species which was subsequently used to calculate dN_{wth}, dS_{wth} and dN_{wth}/dS_{wth} (horizontal axis in (A), (B) and (C), respectively). To confirm the robustness of these evolutionary rates, we alternatively computed the trees for a subset of genes based on a maximum likelihood (ML) method. To this end, a hundred genes were randomly selected, and subjected to the phylogenetic analysis with RAxML (model: GTRGAMMA; bootstrap: 100). Using these trees, we calculated the evolutionary rates (vertical axis), and compared them with that from the NJ method. The solid lines represent y=x. High correlation coefficients (Spearman's rank) indicate a good agreement between the two methods.
